# Extraction and Characterization of Bioactive Fish By-Product Collagen as Promising for Potential Wound Healing Agent in Pharmaceutical Applications: Current Trend and Future Perspective

**DOI:** 10.1155/2022/9437878

**Published:** 2022-05-06

**Authors:** Siti Nur Hazwani Oslan, Cheng Xue Li, Rossita Shapawi, Ruzaidi Azli Mohd Mokhtar, Wan Norhana Md. Noordin, Nurul Huda

**Affiliations:** ^1^Faculty of Food Science and Nutrition, Universiti Malaysia Sabah, Jalan UMS, Kota Kinabalu, 88400 Sabah, Malaysia; ^2^Borneo Marine Research Institute, Universiti Malaysia Sabah, Jalan UMS, Kota Kinabalu, 88400 Sabah, Malaysia; ^3^Biotechnology Research Institute, Universiti Malaysia Sabah, Jalan UMS, Kota Kinabalu, 88400 Sabah, Malaysia; ^4^Fisheries Research Institute, Batu Maung, 11960 Penang, Malaysia

## Abstract

Collagen is a structural protein naturally found in mammals. Vertebrates and other connective tissues comprise about 30% of an animal's overall protein. Collagen is used in a variety of applications including cosmetics, biomedical, biomaterials, food, and pharmaceuticals. The use of marine-based collagen as a substitute source is rapidly increasing due to its unique properties, which include the absence of religious restrictions, a low molecular weight, no risk of disease transmission, biocompatibility, and ease of absorption by the body system. This review discusses recent research on collagen extraction from marine-based raw material, specifically fish by-products. Furthermore, pretreatment on various sources of fish materials, followed by extraction methods, was described. The extraction procedures for acid soluble collagen (ASC) and pepsin soluble collagen (PSC) for fish collagen isolation are specifically discussed and compared. As a result, the efficacy of collagen yield was also demonstrated. The recent trend of extracting fish collagen from marine biomaterials has been summarized, with the potential to be exploited as a wound healing agent in pharmaceutical applications. Furthermore, background information on collagen and characterization techniques primarily related to the composition, properties, and structure of fish collagen are discussed.

## 1. Introduction

Collagen is a structural protein naturally found in mammals, accounting for approximately 30% of total protein in the various connective tissues of an animal's body [1–3]. Collagens derived from bovine, pig, and poultry sources are the most commonly used and commercially viable in the production of collagen products [4]. However, the use of bovine and pig collagens is not acceptable by some religious and ethnic groups. There are also infectious and contagious diseases associated with pigs and cattle, such as Bovine Spongiform Encephalopathy (BSE). Therefore, the applications of animal-derived collagen are frequently contentious and limited [1, 2]. Collagen is essential for structural support and tissue development. Through mechanochemical transduction processes, it ensures tensile strength, firmness, and elasticity of tissues for locomotion, regeneration, and maintenance as structural support [2, 5]. Additionally, it promotes the formation of fibroblasts, a fibrous network of cells that acts as a scaffold for the growth of new cells into cartilages, bones, tendons, and skin tissues with distinct physiological functions [5, 6]. Collagen is used in a wide variety of industries, including food, biomaterials, cosmetics, biomedical, and pharmaceutical such as wound dressing for the treatment of various refractory wounds [1, 4, 5, 7]. The unique functional as a wound healing agent, collagen can protect and accelerates wound healing by preventing pathogen absorption and spread, particularly of harmful microorganisms [8].

In order to meet the increased need for collagen, several researchers have been investigating into alternative sources of collagen, such as marine sources, particularly fish by-products [3, 9]. Many researchers are interested on it because of its ease of extraction, low molecular weight, and lack of hazards from animal diseases and pathogen [1, 2]. Moreover, collagen derived from fish by-products has high biodegradability, biocompatibility, and antigenicity [5]. Furthermore, because of the high daily consumption of fish and the vast number of by-products created each year, which totals more than 20 million tones including the head, fins, skin, and viscera, are discarded as “waste” [6, 10]. Therefore, more study is being undertaken on the extraction method and characterization of collagen form from the fish by-product, such as Nile tilapia (*Oreochromis niloticus*) [3], *Melanogrammus aeglefinus* [11], Amur sturgeon (*Acipenser schrenckii*) [12], silver catfish (*Pangasius* sp.) [13], black ruff (*Centrolophus niger*) [9], sole fish (*Aseraggodes umbratilis*) [14], *Catla catla*, and *Cirrhinus mrigala* [15]. The goal of this review is to provide a current overview of collagen production from various fish uses in medical and pharmaceutical fields, with a focus on extraction and characterization methods that are particularly relevant to the content, properties, and structure of collagen from fish. This review potentially leads to additional discoveries on collagen's potential as a wound healing agent in biomedical applications.

## 2. Collagen Molecular Structure

There are 28-29 varieties of collagens with various structures, sequences, functions, and molecular features in vertebrate tissues, which are coded by at least 45 distinct genes [2, 3]. A multicollagen molecule can produce more collagen than a single collagen molecule that is 300 nm long and 1.5–2.0 nm in diameter [16]. Collagen is made up of basic subunit called “tropocollagen” [17]. It had the characteristic three-dimensional triple helical tertiary structure where the three polypeptide chains are arranged in ideal geometry that maximise structural stability through intrachain hydrogen bonding in relation to the number of hydroxyproline and proline [3, 18]. The collagen family can be divided into several categories due to its significant structural variety. In addition, type I found in connective tissues like skin, bone, and tendons, type II in cartilage tissue, type III in muscle tissue, and other types found in very small volumes and mostly organ-specific [17]. Collagen is made up of all 20 amino acids. Collagen of mammalian origin includes a high concentration of imino acids, particularly hydroxyproline and hydroxylysine and proline and hydroxyproline [18]. Type I collagen, a fibrous collagen with a triple helical structure, is the most prevalent type of collagen found in mammals and fish [19, 20]. It is also the most abundant type collagen found in fish skin, bone, scales, and fins [6]. Fish collagens from various species have been demonstrated to differ significantly in their amino acid content, notably the amounts of proline and hydroxyproline. Furthermore, the composition of amino acids is altered by environmental factors such as temperature. It has been discovered that collagen obtained from warm-water fish species has greater thermal stability than collagen derived from cold-water fish species [18]. Thermal stability differences are also reported in warm-blooded mammals and cold-blooded fishes. This could be related to the content or concentration of the imino acid hydroxyproline, which played a role in interchain hydrogen bonds that contributed to stabilize helix structure of collagen. This role of hydroxyproline, as well as its concentration, has an effect on the thermal stability of fish collagen [21]. Therefore, collagen produced by fish raised in warmer climates, such as tilapia, will contain more imino acids (proline and hydroxyproline) than collagen produced by cold-water fish, such as cod [22, 23]. Tuna had a proline and hydroxyproline content of 230 residues/1000 residues [24], and grass carp had a proline and hydroxyproline content of 161–181 residues/1000 residues [25]. Furthermore, the scale of Rohu and Catla produces a 5% yield and a high imino acid content, resulting in a relatively higher thermal stability collagen at Td 36.5°C [26]. In another study, fish scale collagens from seabass (*Lates calcarifer*) showed a high denaturation temperature (38.17 and 39.32°C) that could be explained by the high amino acid composition, which facilitates the formation of stronger intra/interchain bonds [6, 21].

### 2.1. Marine Collagen Source

In recent studies, it has been discovered that collagen derived from marine resources could be the most convenient and risk-free method of obtaining high-quality collagen [18, 27]. Along with its low molecular weight, it promotes rapid bloodstream circulation and good absorption into the body by up to 1.5 times [1]. Furthermore, according to current research trends in the biomedical field of tissue engineering, collagens of marine origin outperform mammalian collagens and other alternative sources in biomaterial applications due to their significant biocompatibility and biodegradability [28–30]. Marine collagens can currently be obtained from a variety of fish by-products. By-products such as fishbone, scales, skins, and fins have been extensively researched and exploited as a potential alternative for collagen recovery via a combination of different bioprocessing methods [15, 31]. Furthermore, the amino acid composition and biocompatibility of marine source collagen are found similar to those of bovine and porcine collagens [32]. As previously discussed, the majority of fish collagen contains less proline and hydroxyproline than mammalian-based collagen, which results in decreased cross-linking compatibility and stability [18, 27, 33]. Consequently, fish skin is extremely pure (approximately 70%) and is predominantly composed of type I collagen, depending on the species, age, and season [34]. Numerous researchers discovered that collagen production using of fish tilapia skin could yield a dry weight yield of more than 40%, which is highly promising and competitive in the industrial production of collagen peptides [3, 30]. Collagen derived from fish skin has a high water retention capacity (about 6% of its weight after 24 hours of exposure to 63% humidity), and it has no irritant potential, making it appropriate for dermal applications [35]. Collagen extraction yields (4.76 and 8.14 wt%) from Nile tilapia (*Oreochromis niloticus*) skin fermentation-ASC (FASC) and fermentation-PSC (FPSC) were slightly but not significantly greater than chemical-ASC (CASC) and chemical-PSC (CPSC) yields (4.27 and 7.60 wt%), respectively [3]. These collagens denatured between 36.5 and 37.1°C and were highly soluble at pH 1–4 and 3% (*w*/*v*) NaCl. The removal of the nonhelical region by pepsin may have had an effect on the collagen amino acid sequence [36], where the number of imino acids is critical for collagen stability [27].

In other work, the extracted collagen uses acid soluble collagen (ASC) from snakehead fish by-products, including skin and a mixture of skin and scale, yielding 13.6% and 12.09%, respectively [23]. Collagen extracted from the skin various species such as catfish, pomfret, and mackerel requires a slightly lower extraction temperature than 13.26°C, with a longer extraction time (74 h), and produced 2.27% extraction yields [37]. For instance, fish scales are made of extracellular matrix, which demonstrated good water absorption and retention by 13.3% and 15%, respectively, and composed of two 1 chains, one 2 chain, and hydroxyapatite, which combine to form highly ordered collagen fibres with tightly cross-linked regions [2, 38]. Furthermore, collagen extracted from silver carp fish scales produced successful yields (5.09 and 12.06%) when ASC and PSC were used [8]. Consequently, a complex tissue, bone, is composed of a fibrous type I collagen composed of two 1 and one 2 chains accumulating hydroxyapatite (HA) crystals ((Ca)10 (PO4)6(OH)2) [2, 37]. Recently, collagen derived from fishbones of *Lutjanus* sp. was obtained using a hydroextraction method, which yielded 4.535% of the total extract, with a protein concentration of 8.815 mg/mL [39].

## 3. Collagen Extraction Methods

### 3.1. Pretreatment

Recent research shows the use of various collagen extraction methods on various parts of aquatic animals such as skin, scale, bone, or cartilage. The general procedure for collagen isolation begins with preparation, then moves on to extraction, and finally to recovery [2]. Collagen production consists primarily of pretreatment, extraction, separation, purification, and characterization [40]. The first step is known as preparation, and it varies depending on the type of raw materials used. To reduce sample contamination, pretreatment procedures such as washing, cleaning, and size reduction are required prior to extraction [41]. Acidic and alkali pretreatments are also commonly used to remove noncollagenous substances such as proteins, fats, and pigments in fish in order to achieve higher purity while maximising the yield and quality of the extracted collagen. A mild chemical solution of diluted acid or base is frequently used to degrade cross-linked collagen in animal connective tissue [42]. For example, acidic pretreatment is appropriate for raw materials containing collagen with fewer cross-links, as it allows noncovalent bonds to be broken under controlled temperature. The raw materials are immersed in a solution of acid. On the other hand, alkaline pretreatment is typically used to depolymerize the inter- and intramolecular cross-links in thick, hard raw material in order to remove noncollagenous substances [42, 43]. Sodium hydroxide, calcium hydroxide, hydrogen peroxide, butyl alcohol, salt, alkali, and a combination of alkali and detergent are currently used in common pretreatment methods. It is necessary to treat collagen extraction raw materials like skin or scales before using them. A combination of various degreasing agents is also used sequentially [41, 44].

In addition, when performing alkaline pretreatment with sodium hydroxide (NaOH), a concentration range of NaOH (0.05–0.10 M) can be used and accommodated [42]. NaOH is widely used because of its higher swelling ability, which can aid in the extraction of collagen by maximising the rate of mass transfer within the tissue matrix [25]. Furthermore, pretreatment with a hydrogen peroxide solution (3% (*w*/*v*), pH 10) prior to collagen extraction had a significant impact on the structure of the collagen produced particularly for removing snakehead skins [41]. Therefore, selection of an appropriate method for pretreatment of raw material is needed to avoid generating excessive waste liquid and forming reagent residues in collagen, resulting in environmental pollution [3]. Another pretreatment, known as the demineralization process, is used to improve the efficiency of collagen extraction. It is used in the extraction of collagen from raw materials that contain of minerals including bone and scales. The ethylenediaminetetraacetic acid (EDTA) is used through demineralization process [43]. EDTA acts as a calcium ion chelating agent by increasing the utilization of the substrate [42].

### 3.2. Extraction Method

Currently, there are many procedures available today that allow for better retention of the substance such as collagen, with a focus on the extraction methods to achieve higher collagen yields, primarily from fish materials. The method used to extract collagen has an impact on the amount of collagen produced and its properties [13]. Chemical collagen extraction methods which use alkali and acid and biochemical collagen extraction methods which use enzymes are both available using acid and enzyme or acid and microorganisms [34]. In addition to the extraction methods, ultrafiltration and homogenization are used to keep the collagen triple-helix structure, batch-to-batch yields, and quality consistent from batch to batch [3]. For instance, the most popular method of collagen extraction involves using acid and enzyme [1, 13, 45–47]. Organic acids such as chloracetic, citric, or lactic acid can be used to extract collagen from cells using the acid method [13]. Based on Table 1, the acid soluble collagen extraction and the pepsin-solubilized collagen extraction method are the most commonly used collagen extraction methods.

#### 3.2.1. Acid-Solubilized Collagen (ASC) Extraction Procedure

Acid-solubilized collagen (ASC) is a type of collagen that is extracted solely with acid. The acid-collagen reaction breaks cross-links in helix of the collagen, enhancing the quality of collagen extracted. Because of this, the use of various acids to maximise extraction efficiency, purity, and yield of collagen has always been an area of study. Based on collagen extraction of silver catfish skin, it was discovered that by using 0.5 M acetic acid and a sample to solution ratio of 1 : 10 (*w*/*v*), a yield of 4.270.06% could be obtained after 24 hours of incubation [13]. Recently, Kuwahara [27] has been reported that the use of ultrafine bubbles of carbon dioxide in a mild 0.1 M acetic acid solution for 5 hours can be considered as the most effective method to extract collagen from tilapia scales with the yield of 1.58%. The interaction of collagen molecules with the AcOH solution improved the collagen yield from 0.518 to 1.581 mg/mL when the AcOH concentration was increased from 0.1 to 2.0 M [48]. This was supported by the findings of other study on extraction of collagen from the skin of sole fish using AcOH at concentrations ranging from 0.2 to 1.0 M; resulted of using 0.6 M of AcOH, the yield of collagen was 15.968 mg/g [14]. In addition, Truong et al. [23] revealed that yield of fish skin recovered from snakeheads was 13.6%, and scale was 12.09% via ASC extraction technique.

#### 3.2.2. Pepsin Soluble Collagen (PSC) Extraction Procedure

Generally, another method of collagen extraction that is frequently used is pepsin soluble collagen (PSC) extraction, where pepsin is added into the extraction process [24]. According to Yu et al. [49], three critical parameters for the PSC extraction method were optimized: the best conditions for the PSC extraction method are a pepsin concentration of 1389 U/g, a solid-liquid ratio of 1 : 57, and a hydrolysis time of 8.67 hours With these parameters, the PSC extraction yield could reach 84.85%. According to Song et al. [3], collagen was extracted from the skin of the Nile tilapia using a fermentation method (FPSC) and a chemical method (CPSC) pretreatment. Raw skin materials were embedded in 0.5 M acetic acid at a 1 : 80 (*w*/*v*) ratio, containing 5% pepsin (*w*/*w*) for 24 hours. The yield of FPSC and CPSC was 8.14 and 7.60%, respectively. In addition, the yield of collagen from the mackerel skin extracted using PSC method was 8.10% [50]. Besides that, it was reported by a previous study that collagen was extracted from five different tissues of bighead carp fins and scales, skins and bones, and swim bladders through PSC after they were treated with 0.1 M NaOH and alkaline solution [51]. Skins and swim bladders yielded significantly more collagen (*P* < 0.05) than fins, scales, and bones, which yielded 5.1, 2.7, 60.3, 2.9, and 59.0%, respectively. The variation in extracted collagen yield could be attributed to differences in fish species, raw materials, pretreatment, and extraction method [3].

#### 3.2.3. Ultrasound Extraction Methods

Ultrasound was previously reported as a method for increasing the efficiency of collagen extraction from fish materials. However, the downside of this method is that ultrasound could negatively impact the physicochemical and molecular characteristics of collagen [45]. The ultrasound-assisted (UA) extraction of collagen from clown featherback (*Chitala ornata*) skin has been explored by Petcharat et al. [45] using different amplitudes (20–80%) and duration (10–30 min). This significantly increases the yield of collagen (*P* < 0.05), which is from 27.18 to 57.35%. However, the extraction by using UA treatment might influence collagen purity due to the decreasing hydroxyproline content. This could be caused by induced protein degradation, which would be particularly apparent with increasing amplitude and duration. In addition, the other study had reported that the yield of collagen from sea bass skin reached 90.40% after exposed to 24 hours of UA treatment with 0.1 M acetic acid and an ultrasonic frequency of 20 kHz with amplitudes of 80% [54]. Therefore, the most effective duration time under UA was 3 hours. It was showed the collagen extraction by the ultrasonic treatment did not cause changes in the main components after pepsin treatment. As a consequence, the use of ultrasonication under the right conditions has a high potential for enhancing the extraction yield of collagen from fish materials. Furthermore, it may enhance the cavitation effect by disrupting the cell walls in skin tissue, resulting in the liberation of collagen [55].

The other method of extraction of collagen is the extrusion-hydro-extraction (EHE) process. According to Huang et al. [38], the EHE process produced a 2–3 times higher protein extraction yield from tilapia (Oreochromis sp.) fish scales than nonextruded scale samples, with hydroxyproline (61–73 residues/1000 residues) and hydroxylysine (5–6 residues/1000 residues) found in all extracts. Other than improving the yield, EHE process also offers other advantages like possibility for continuous production, simple in operation, and minimal waste production. Other reactions such as thermal treatment, protein denaturation, and mashing are typical reactions that follow the extrusion process [2]. Table 1 summarizes articles on current collagen extraction methods for isolating collagen from fish by-products.

## 4. Collagen Characterization Methods

Characterization of isolated collagen establishes a logical link between result and structural characteristics. A variety of techniques can be used to characterize marine collagen, particularly its structure and morphological properties. In collagen characterization, Fourier transform infrared (FTIR) spectroscopy is a widely used technique for analyzing the secondary structure of proteins. FTIR spectroscopy is relevant because it reveals each absorption wavenumber in the spectrum between 500 and 4000 cm^−1^ [56, 57]. The amide band FTIR spectroscopy confirmed the presence of amide A, B, I, II, and III bands in collagen extracted from bones of *Lutjanus* sp., where amide III showed the triple helix in extracted collagen [39]. Other studies have used FTIR to determine the type and chemical composition of collagen as well as the presence of collagen. For example, Asaduzzaman et al. [50] use FTIR spectra to characterize PSCs extracted from mackerel (*Scomber japonicus*) bones and skin. The findings revealed that type I collagen, a heterotrimer containing two identical *α*1-chains and one *α*2-chain in the molecular form, was primarily constituent of mackerel bones and skin. Furthermore, the FTIR spectra of collagens extracted from seabass scales using ASC and PSC extraction procedures, with the results indicating that the collagen is type I and the functional groups in the triple helix have not been damaged by the treatment process [6]. Furthermore, the majority of the collagen from marine species was discovered to be type I, and FTIR analysis has been shown in numerous studies to be an effective structural characterization method for collagen. In addition, SDS-PAGE (sodium dodecyl sulphate polyacrylamide gel electrophoresis) is a technique that has been used for separating protein and protein fragments based on size. SDS-PAGE can be used to determine the molecular weight of collagen [58]. When the collagen bands are similar, it is possible to determine the type of collagen by comparing it to collagen from other sources. Collagen is composed of three *α* chains that can be identical or dissimilar depending on the type of collagen. According to Arumugam et al. [14], the collagen extracted from the skin of sole fish contained three -chains, namely, (*α*1)_2_, *α*2 (M.W. 118, 116 kDa), and one *β* chain (M.W. 200 kDa). Furthermore, circular dichroism (CD) spectrometry also can be used to determine the secondary structure, folding, and binding properties of collagen proteins. CD can reveal form of protein structure in extracted collagen including its denatured form–coil structure or triple helical structure. Collagen is composed of three alpha chains, the number of which varies according to the type of collagen [59]. According to Petcharat et al. [45], all collagen extracted from crown featherback (*Chitala ornata*) skin using an ultrasound-assisted extraction method exhibited triple-helical structure characteristics by using CD spectrometry. The CD technique, for example, can be viewed as a quick way to investigate the folding of the proteins of the collagen fragments. They can be difficult to fold and must be prefolded for several days on ice or several hours at 25°C before CD analysis. For amino acid analysis, chromatographic techniques can be used in separation, identification, and quantification of amino acids [2]. For example, Wu et al. [8] reported that amino acid analyses from fish scale collagen extracted of silver carp (*Hypophthalmichthys molitrix*) via ASC and PSC were type I collagens with a high glycine content of 34% and a low imino acid content of 20% (Pro and Hyp).

## 5. Application Fish Collagen as Wound Healing

In some burn reactions, the use of synthetic drugs to treat wound healing may result in drug resistance. Furthermore, wound healing entails multiple phases, including coagulation, inflammation, granulation, proliferation, matrix synthesis and deposition, fibrogenesis, angiogenesis, wound contractions, and reepithelization. All of these are challenges to wound healing. As a consequence, it is necessary to investigate medications derived from natural substances. Complications and pain are occasionally exposed due to the slowness with which the damage heals. Due to its biocompatibility, biodegradability, and greater ability to penetrate lipid-free interfaces, collagen has numerous biomedical applications. Importance of collagen in the biomedical field stems from its ability to self-arrange and cross-link of collagen fibres form with high strength and stability [60, 61]. Most researchers have proven that collagen hydrogels are the best candidate materials for wound dressings. This is due to the three-dimensional structure, which is very similar to the moisture at the natural extracellular of skin and ensures that the wounds have the same moist environment [30]. Collagen's excellent properties as a wound healing dressing have been proven, and the entire process has been thoroughly investigated. It is nonantigenic, sensitive to gaseous exchange, and impermeable to bacterial contamination. Furthermore, flexibility and elasticity of collagen allow it to conform well to underlying topography while reducing pain and healing time and increasing long-term aesthetics. Furthermore, collagen is involved in the formation of extracellular matrix (ECM) as well as the development/migration of cells and tissues. Apart from that, it can stimulate and promote the production of keratinocytes and fibroblasts near the wound, which can improve the healing process significantly. For example, collagen extracted from fish has anti-inflammatory and immune-modulatory properties, as well as antimicrobial activity and the ability to improve wound healing [5, 60].

Elbialy et al. [30] investigated the ability of collagen extracted from Nile tilapia (*Oreochromis niloticus L.*) skin to improve cutaneous wound healing in rats. The study employs a rat model that is under general anesthesia and has full-thickness wounds on its back. Following that, the wound was topically treated with tilapia collagen extract. This study discovered that applying tilapia collagen to a wound stimulated healing by increasing keratinocyte proliferation, fibroblast and myofibroblast differentiation, and ECM production. According to Shalaby et al. [60], the purpose of this study was to explore the influence of tilapia and grey mullet collagen on wound healing in rats. The results obtained demonstrated that increasing cell adhesion capacities improves wound healing, resulting in improved wound resolution and closure. Moreover, hydroxyproline is a particular element of the protein collagen, and its concentration could be utilized as an indicator to estimate collagen deposition and thus the efficiency of wound healing. Collagens are involved in platelet aggregation, inflammation modulation, angiogenesis, the formation of granulation tissue, and wound reepithelialization. These receptors bind matrix-integrated collagen and are involved in the regulation of critical wound healing processes. These signaling molecules' loss of activity reduces keratinocyte growth and collagen remodeling, resulting in weak wounds. However, pathological processes including scar development cause abnormal collagen signaling. As a result of the experiment, it was revealed that fish collagen could be a promising candidate for wound healing applications.  Table 2 summarizes recent articles on the use of collagen extracted from fish by-products for wound healing.

## 6. Current Trends and Future Perspective

Exploration of marine resources, which is a new trend, produces a lot of biowaste that benefits the environment. By-products of marine processing have added value to what was previously classified as biowaste. These often-discarded by-products have been investigated for their potential to yield high-value products such as collagen, enzymes, proteins, and fish oils. Previous research has demonstrated that a component with an excellent potential can be extracted from fish by-products. Additional research is required while the most critical extraction and characterization methods for these products are in limited supply. As a result, the quest for novel approaches for extracting and purifying high-value items from fish waste may represent an ideal opportunity for the fish processing industries to add value to what has already been considered wastes. Collagen derived from fish by-products has attracted biotechnologists' attention, primarily for use as a wound healing agent in biomedicine via collagenous extracts from fish. Despite its numerous applications and potential in tissue engineering, collagen is a critical biopolymer for tissue regeneration. For instance, the demand and need for a more sustainable and cost-effective source of bioactive peptide collagen production become even more critical. The different technique of fish collagen extraction methods resulted the various of collagen yield and compositions. The temperature, extraction time, and solvent concentration, all have an effect on collagen extraction methods. The yield of collagen can be increased by optimizing the extraction process. It is suggested that future research look into the structure of marine collagen by using the novel enzymatic and chemical technique. This may improve the suitability of marine collagen as a wound healing. Furthermore, collagen extraction is crucial in medical tissue, particularly in bone tissue engineering, cartilage tissue engineering, and skin tissue functional restoration. It is the greatest template for cell growth due to its high biocompatibility. However, there are not many types of collagen on the market right now. As a result, the application potential of all types of collagen is vast. Collagen derived from fish has gained widespread recognition as a novel biomaterial, attracting increasing interest from clinical, medical, food, and other researchers. Furthermore, thermal or acid/base treatments can also convert collagen to gelatin. Acid, alkali, proteolytic enzymes, or heat converts collagen's insoluble fibrous structure into water-soluble gelatin. Gelatin is widely used in food, medicine, cosmetics, and photography.

## 7. Conclusions

According to current research, a particularly promising source of natural collagen for nonporcine and nonbovine sources, marine fish collagen has the potential to satisfy the demands for natural collagen from nonporcine and nonbovine sources. Among the available alternatives, fish can be considered the best source of raw material for collagen extraction due to its high availability, lack of disease transmission risk, lack of religious barriers, and potential for higher yielding collagen. Therefore, fish by-products are becoming a more popular alternative source for collagen extraction.

## Figures and Tables

**Table 1:** Summary of fish collagen productivity by different extraction method and characterization of isolation of collagen from various species of fish.

Fish species	Source collagen	Extraction method	Characterization	Productivity	References
Clown featherback (*Chitala ornata*)	Skins	Acid soluble collagen (ASC) assisted with ultrasonication treatment	Triple-helical structure—type I collagen	Ultrasonication treatment increases collagen extraction yield (27.18-57.35%) compared to standard process (23.46%)	[45]
Snakeheads (*Channa striata*)	Skin, scales	Acid soluble collagen (ASC)	Type I	The recovery yield of fish skin (13.6%) was significantly greater than the recovery yield of a mixture of fish skin and scale (12.09%)	[23]
*Prionace glauca*, *Scyliorhinus canicula*, *Xiphias gladius*, and *Thunnus albacares*	Skin	Pepsin soluble collagen (PSC)	Type I	Collagen yield was obtained from the skin of two species of teleost, and chondrichthyes were 14.16% and 61.17%, respectively	[52]
Tilapia (*Oreochromis niloticus*)	Scales	Acetic acid and ultrafine bubbles	Type I	Scales were used to extract collagen, which resulted in a yield of 1.58%	[27]
Lutjanus sp.	Bone	Hydroextraction method	Triple-helical structure—type I collagen	In this experiment, the collagen yield was 4.535%, with a protein concentration of 8,815 mg/mL	[39]
Silver carp (*Hypophthalmichthys molitrix*)	Scales	Acid soluble collagen (ASC); pepsin soluble collagen (PSC)	Triple-helical structure—type I collagen	The collagen extraction yields of ASC and PSC were 5.09% and 12.06%, respectively	[8]
Seabass (*Lates calcarifer*)	Scales	Acid soluble collagen (ASC); pepsin soluble collagen (PSC)	Triple-helical structure—type I collagen	Yields of collagen from ASC and PSC were 0.38 and 1.06%, respectively	[6]
Nile tilapia (*Oreochromis niloticus*)	Skin	Acid soluble collagen (ASC); pepsin soluble collagen (PSC)	Type I collagen with triple-helical structure	Fermentation-ASC (FASC) and fermentation-PSC (FPSC) yields were 4.76 and 8.14 wt%, respectively; chemical-ASC (CASC) and chemical-PSC (CPSC) yields were 4.27 and 7.60 wt%, respectively	[3]
Amur sturgeon (*Acipenser schrenckii*)	Cartilage, fin and scale	Salt-soluble collagen, (SSC); acid soluble collagen (ASC); pepsin soluble collagen (PSC)	Type I collagen SSC and ASC; PSC predominantly type II with triple helical structure.	Yields of collagen from SSC (2.18%), ASC (27.04%), and PSC (55.92%), respectively	[53]
Silver catfish (*Pangasius* sp.)	Skin	Acid soluble collagen (ASC) and pepsin soluble collagen (PSC)	Isolated collagens exhibited amide A, II, and III as a fingerprint for collagen structure	Yields of collagen from ASC and PSC were 4.27% and 2.27%, respectively. ASC and PSC had protein concentrations of 2.27 and 2.70 mg/mL, respectively	[13]
Sole fish (*Aseraggodes umbratilis*)	Skin	Acid soluble collagen (ASC)	Type I collagen with triple-helical structure	The maximum yield of collagen was 19.27 mg per gram of skin and achieved at optimum conditions	[14]
Small-spotted catshark (*Scyliorhinus canicula*)	Skins	Acid soluble collagen (ASC)	Type I collagen with triple-helical structure	The maximum collagen yield was 61.24%	[40]
Tilapia (*Oreochromis mossambicus*)	Bone	Desalinated with EDTA-ASC; EDTA-PSC; desalinated HCl-PSC	Type I collagen which corresponding with secondary structure	Yield of collagen desalinated with EDTA − ASC = 2.5%; desalinated with EDTA − PSC = 7.3%; desalinated by HCl − PSC = 0.5%	[37]

**Table 2:** Summary of recent extraction of fish collagen for wound healing application.

Fish species	Source collagen	Remarks	References
Nile tilapia (*Oreochromis niloticus L.*)	Skin	Collagen extraction can increase TGF-*β*1, b-fibroblast growth factor (b-FGF), *α*-smooth muscle actin (*α*-SMA) gene expression, fibroblast and myofibroblast proliferation, and ECM production	[30]
Snakehead fish (*Channa striata*)	Skin	New dressing for burn healing has the potential to be used by cross-linking biopolymer collagen with alginate to form functional group –CONH	[62]
Tilapia and grey mullet	Scale	Through self-aggregation and cross-linking, all of the extracted collagen forms fibres with increased strength and stability, which help maintain optimal moisture levels at the wound site, which promotes wound healing, and have inhibitory activity against all tested bacteria	[60]
*Prionace glauca*, *Scyliorhinus canicula*, *Xiphias gladius*, and *Thunnus albacares*	*Skin*	Collagen extraction significantly accelerated the healing of deep second-degree burn wounds and the generation of new skin appendages that can be used to treat a variety of refractory wounds, according to skin repair experiments	[52]
*Melanogrammus aeglefinus*	Skin	The experiments revealed that by extracting collagen from fish skin and testing it on mice, fibrin was formed, resulting in a decrease in clotting time, which could accelerate epithelialization and shorten the wound healing time of mice and thus shorten the bleeding times	[11]

## Data Availability

Data used and/or analyzed in the study are available from the corresponding author on reasonable request.

## References

[1] Senadheera T. R. L., Dave D., Shahidi F. (2020). Sea cucumber derived type I collagen: a comprehensive review. *Marine Drugs*.

[2] Jafari H., Lista A., Siekapen M. M. (2020). Fish collagen: extraction, characterization, and applications for biomaterials engineering. *Polymers*.

[3] Song Z., Liu H., Chen L. (2021). Characterization and comparison of collagen extracted from the skin of the Nile tilapia by fermentation and chemical pretreatment. *Food Chemistry*.

[4] Huda N., Seow E. K., Normawati M. N., Nik Aisyah N. M., Fazilah A., Easa A. M. (2013). Effect of duck feet collagen addition on physicochemical properties of surimi. *International Food Research Journal*.

[5] Ge B., Wang H., Li J. (2020). Comprehensive assessment of Nile tilapia skin (*Oreochromis niloticus*) collagen hydrogels for wound dressings. *Marine Drugs*.

[6] Chuaychan S., Benjakul S., Kishimura H. (2015). Characteristics of acid- and pepsin-soluble collagens from scale of seabass (*Lates calcarifer*). *LWT - Food Science and Technology*.

[7] Kan M., Qian X., Zhang T., Yue D., Zhao Y. (2017). Highly active IrO_x_ Nanoparticles/Black Si electrode for efficient water splitting with conformal TiO_2_ interface engineering. *ACS Sustainable Chemistry & Engineering*.

[8] Wu J., Kong L., Zhang J., Chen W. (2019). Extraction and properties of acid-soluble collagen and pepsin-soluble collagen from silver carp (*Hypophthalmichthys molitrix*) scales: prerequisite information for fishery processing waste reuse. *Polish Journal of Environmental Studies*.

[9] Bhuimbar M. V., Bhagwat P. K., Dandge P. B. (2019). Extraction and characterization of acid soluble collagen from fish waste: development of collagen-chitosan blend as food packaging film. *Journal of Environmental Chemical Engineering*.

[10] Caruso G. (2015). Fishery wastes and by-products: a resource to be valorised. *Journal of Fisheries Sciences*.

[11] Dang Q. F., Liu H., Yan J. Q. (2015). Characterization of collagen from haddock skin and wound healing properties of its hydrolysates. *Biomedical Materials (Bristol)*.

[12] Wang L., Liang Q., Chen T., Wang Z., Xu J., Ma H. (2014). Characterization of collagen from the skin of Amur sturgeon (*Acipenser schrenckii*). *Food Hydrocolloids*.

[13] Hukmi N. M. M., Sarbon N. M. (2018). Isolation and characterization of acid soluble collagen (ASC) and pepsin soluble collagen (PSC) extracted from silver catfish (*Pangasius* sp.) skin. *International Food Research Journal*.

[14] Arumugam G. K. S., Sharma D., Balakrishnan R. M., Ettiyappan J. B. P. (2018). Extraction, optimization and characterization of collagen from sole fish skin. *Sustainable Chemistry and Pharmacy*.

[15] Mahboob S. (2015). Isolation and characterization of collagen from fish waste material- skin, scales and fins of *Catla catla* and *Cirrhinus mrigala*. *Journal of Food Science and Technology*.

[16] Paul R. G., Bailey A. J. (2003). Chemical stabilisation of collagen as a biomimetic. *TheScientificWorldJournal*.

[17] Silva T. H., Moreira-Silva J., Marques A. L. P., Domingues A., Bayon Y., Reis R. L. (2014). Marine origin collagens and its potential applications. *Marine Drugs*.

[18] Shahidi F., Varatharajan V., Peng H., Senadheera R. (2019). Utilization of marine by-products for the recovery of value-added products. *Journal of Food Bioactives*.

[19] Yousefi M., Ariffin F., Huda N. (2017). An alternative source of type I collagen based on by-product with higher thermal stability. *Food Hydrocolloids*.

[20] Yousefi M., Ariffin F., Huda N., Williams P. A., Philips G. O. (2016). Purification and Biochemical Properties of Type I Collagen from Quail Feet (*Coturnix japonica*). *Gum and Stabilizers for Food Industry 18: Hydrocolloid Functionality for Affordable and Sustainable Global Food Solutions*.

[21] Zhang X., Xu S., Shen L., Li G. (2020). Factors affecting thermal stability of collagen from the aspects of extraction, processing and modification. *Journal of Leather Science and Engineering*.

[22] Tziveleka L. A., Ioannou E., Tsiourvas D., Berillis P., Foufa E., Roussis V. (2017). Collagen from the marine sponges *Axinella cannabina* and *Suberites carnosus*: isolation and morphological, biochemical, and biophysical characterization,. *Marine Drugs*.

[23] Truong T. M. T., Nguyen V. M., Tran T. T., Le T. M. T. (2021). Characterization of acid-soluble collagen from food processing by-products of snakehead fish (*Channa striata*). *Processes*.

[24] Ahmed R., Haq M., Chun B. S. (2019). Characterization of marine derived collagen extracted from the by-products of bigeye tuna (*Thunnus obesus*). *International Journal of Biological Macromolecules*.

[25] Liu D., Wei G., Li T. (2015). Effects of alkaline pretreatments and acid extraction conditions on the acid- soluble collagen from grass carp (*Ctenopharyngodon idella*) skin. *Food Chemistry*.

[26] Pati F., Adhikari B., Dhara S. (2010). Isolation and characterization of fish scale collagen of higher thermal stability. *Bioresource Technology*.

[27] Kuwahara J. (2021). Extraction of type I collagen from tilapia scales using acetic acid and ultrafine bubbles. *Processes*.

[28] Li B. (2017). Beneficial effects of collagen hydrolysate: a review on recent developments. *Biomedical Journal of Scientific & Technical Research*.

[29] Bernhardt A., Paul B., Gelinsky M. (2018). Biphasic scaffolds from marine collagens for regeneration of osteochondral defects. *Marine Drugs*.

[30] Elbialy Z. I., Atiba A., Abdelnaby A. (2020). Collagen extract obtained from Nile tilapia (*Oreochromis niloticus* L.) skin accelerates wound healing in rat model via up regulating VEGF, bFGF, and *α*-SMA genes expression. *BMC veterinary research*.

[31] Dave D., Routray W. (2018). Current scenario of Canadian fishery and corresponding underutilized species and fishery byproducts: a potential source of omega-3 fatty acids. *Journal of Cleaner Production*.

[32] Wu J. J., Woods P. E., Eyre D. R. (1992). Identification of cross-linking sites in bovine cartilage type IX collagen reveals an antiparallel type II-type IX molecular relationship and type IX to type IX bonding. *Journal of Biological Chemistry*.

[33] Berillis P. (2015). Marine collagen: extraction and applications. *Research trends in biochemistry, molecular biology and microbiology*.

[34] Chinh N. T., Manh V. Q., Trung V. Q. (2019). Characterization of collagen derived from tropical freshwater carp fish scale wastes and its amino acid sequence. *Natural Product Communications*.

[35] Cumming M. H., Hall B., Hofman K. (2019). Isolation and characterisation of major and minor collagens from hyaline cartilage of hoki (*Macruronus novaezelandiae*). *Marine Drugs*.

[36] Zhang J., Jeevithan E., Bao B. (2016). Structural characterization, in-vivo acute systemic toxicity assessment and in-vitro intestinal absorption properties of tilapia (*Oreochromis niloticus*) skin acid and pepsin solublilized type I collagen. *Process Biochemistry*.

[37] Liu H., Huang K. (2016). Structural characteristics of extracted collagen from tilapia (*Oreochromis mossambicus*) bone: effects of ethylenediaminetetraacetic acid solution and hydrochloric acid treatment. *International Journal of Food Properties*.

[38] Huang C.-Y., Kuo J.-M., Wu S.-J., Tsai H.-T. (2016). Isolation and characterization of fish scale collagen from tilapia (*Oreochromis* sp.) by a novel extrusion–hydro-extraction process. *Food Chemistry*.

[39] Ramli L., Natsir H., Dali S., Danial S. (2019). Collagen extraction from bone of Lutjanus sp. and toxicity assay. *Jurnal Akta Kimia Indonesia (Indonesia Chimica Acta)*.

[40] Blanco M., Vázquez J. A., Pérez-Martín R. I., Sotelo C. G. (2019). Collagen extraction optimization from the skin of the small-spotted catshark (*S. canicula*) by response surface methodology. *Marine Drugs*.

[41] Liu W., Zhang Y., Cui N., Wang T. (2019). Extraction and characterization of pepsin-solubilized collagen from snakehead (*Channa argus*) skin: effects of hydrogen peroxide pretreatments and pepsin hydrolysis strategies. *Process Biochemistry*.

[42] Schmidt M. M., Dornelles R. C. P., Mello R. O. (2016). Collagen extraction process. *International Food Research Journal*.

[43] Pal G. K., Suresh P. V. (2016). Sustainable valorisation of seafood by-products: recovery of collagen and development of collagen-based novel functional food ingredients. *Innovative Food Science and Emerging Technologies*.

[44] Tan Y., Chang S. K. C. (2018). Isolation and characterization of collagen extracted from channel catfish (*Ictalurus punctatus*) skin. *Food Chemistry*.

[45] Petcharat T., Benjakul S., Karnjanapratum S., Nalinanon S. (2021). Ultrasound-assisted extraction of collagen from clown featherback (*Chitala ornata*) skin: yield and molecular characteristics. *Journal of the Science of Food and Agriculture*.

[46] Ibáñez E., Mendiola J. A., Castro-Puyana M. (2016). Supercritical Fluid Extraction. *Encyclopaedia of Food and Health*.

[47] Bai C., Wei Q., Ren X. (2017). Selective extraction of collagen peptides with high purity from cod skins by deep eutectic solvents. *ACS Sustainable Chemistry & Engineering*.

[48] Yang H., Duan L., Li Q., Tian Z., Li G. (2018). Experimental and modelling investigation on the rheological behavior of collagen solution as a function of acetic acid concentration. *Journal of the Mechanical Behavior of Biomedical Materials*.

[49] Yu F., Zong C., Jin S. (2018). Optimization of extraction conditions and characterization of pepsin-solubilised collagen from skin of giant croaker (*Nibea japonica*). *Marine Drugs*.

[50] Asaduzzaman A. K. M., Getachew A. T., Cho Y. J., Park J. S., Haq M., Chun B. S. (2020). Characterization of pepsin-solubilised collagen recovered from mackerel (*Scomber japonicus*) bone and skin using subcritical water hydrolysis. *International Journal of Biological Macromolecules*.

[51] Liu D., Liang L., Regenstein J. M., Zhou P. (2012). Extraction and characterisation of pepsin-solubilised collagen from fins, scales, skins, bones and swim bladders of bighead carp (*Hypophthalmichthys nobilis*). *Food Chemistry*.

[52] Blanco M., Vázquez J. A., Pérez-Martín R. I., Sotelo C. G. (2017). Hydrolysates of fish skin collagen: an opportunity for valorizing fish industry byproducts. *Marine Drugs*.

[53] Liang Q., Wang L., Sun W., Wang Z., Xu J., Ma H. (2014). Isolation and characterization of collagen from the cartilage of Amur sturgeon (*Acipenser schrenckii*). *Process Biochemistry*.

[54] Kim H. K., Kim Y. H., Park H. J., Lee N. H. (2013). Application of ultrasonic treatment to extraction of collagen from the skins of sea bass *Lateolabrax japonicus*. *Fisheries Science*.

[55] Sae-leaw T., Benjakul S. (2018). Antioxidant activities of hydrolysed collagen from salmon scale ossein prepared with the aid of ultrasound. *International Journal of Food Science & Technology*.

[56] Abraham L. C., Zuena E., Perez-Ramirez B., Kaplan D. L. (2008). Guide to collagen characterization for biomaterial studies. *Journal of Biomedical Materials Research Part B: Applied Biomaterials*.

[57] Dong X., Shen P., Yu M., Yu C., Zhu B., Qi H. (2020). (−)-Epigallocatechin gallate protected molecular structure of collagen fibers in sea cucumber *Apostichopus japonicus* body wall during thermal treatment. *LWT*.

[58] León-López A., Morales-Peñaloza A., Martínez-Juárez V. M., Vargas-Torres A., Zeugolis D. I., Aguirre-Álvarez G. (2019). Hydrolyzed collagen-sources and applications. *Molecules*.

[59] Bielajew B. J., Hu J. C., Athanasiou K. A. (2020). Collagen: quantification, biomechanics and role of minor subtypes in cartilage. *Nature Reviews Materials*.

[60] Shalaby M., Agwa M., Saeed H., Khedr S. M., Morsy O., El-Demellawy M. A. (2020). Fish scale collagen preparation, characterization and its application in wound healing. *Journal of Polymers and the Environment*.

[61] Stoica A. E., Chircov C., Grumezescu A. M. (2020). Hydrogel dressings for the treatment of burn wounds: an up-to-date overview. *Materials*.

[62] Issains F. B., Trinanda A. F., Basyir A. M., Benaya A., Yuwono A. H., Ramahdita G. (2019). Extraction of collagen Type-I from snakehead fish skin (*Channa striata*) and synthesis of biopolymer for wound dressing. *AIP Conference Proceedings*.

